# Do new quality productivity forces contribute to wellness tourism resilience? Empirical evidence from China

**DOI:** 10.3389/fspor.2025.1618562

**Published:** 2025-10-07

**Authors:** Heng Wei, Yitong Zhang, Guo Hu

**Affiliations:** ^1^ School of Economics and Management, Shanxi University, Taiyuan, China; ^2^ Department of Tourism Culture and Art, Shanxi Vocational College of Tourism, Taiyuan, China

**Keywords:** new quality productivity forces, wellness tourism, human capital aggregation, double-Threshold effect, industrial resilience

## Abstract

Building upon the theoretical foundation of New Quality Productivity Forces (NQPF) and its integration with industrial applications, this study takes wellness tourism as the research carrier and constructs the theoretical framework of “three-dimensional empowerment and four-dimensional evaluation”. Methodologically, We employ an integrated approach combining ArcGIS 10.8 spatial analysis technology with a two-way fixed effects model to empirically examine the spatiotemporal evolution characteristics and driving mechanisms of wellness tourism industries across 30 Chinese provinces from 2014 to 2022. Key findings reveal that: (1) NQPF significantly enhance the resilience of wellness tourism by promoting industrial structure upgrading. (2) Human capital agglomeration, as a critical threshold variable, exhibits a dual-threshold effect on the enabling impact of NQPF, demonstrating a distinct nonlinear leapfrog pattern. This research not only expands the application boundary of NQPF theory in the health field but also provides a reference for the government to formulate health tourism industry policy with both theoretical depth and practical value.

## 1 Introduction

Under the dual challenges of the normalization of global public health crises and the acceleration of population aging, building resilient health ecosystems has become a core proposition in the strategic layout of countries. The European Commission launched the “Action plan 2021–2027 tracker: health”, in March 2021, spending 5.1 billion euros to strengthen cross-border medical collaboration and health emergency response mechanisms. This marks a shift in global health governance from passive crisis response to active resilience building. In this process, wellness tourism has been given a new strategic value due to its unique industrial effect, and is becoming a key factor in accelerating the optimization of the tourism industry structure and the mutual integration of different modes of industry (1). Demand for wellness tourism from the society has certain potential, and countries around the world have taken a series of measures to build a more resilient wellness tourism system Singapore realizes the shift from traditional medical tourism to wellness tourism through the Wellness Tourism Plan, the UAE actively promotes the concept of “wellness tourism”, combining traditional Arabic medicine with modern health care concepts to form a unique spectrum of wellness tourism. These practices show that wellness tourism has gone beyond the realm of purely economic activities and has evolved into a key indicator of regional public health governance capacity and the level of sustainable tourism development.

Chinese society's attention to the wellness tourism industry is continuing to increase, and since the first five “National Wellness Tourism Demonstration Bases” in January 2016, the scale of China's wellness tourism industry has continued to expand, and as of 2020, the number of people traveling for wellness tourism has reached 67.5 million people, its connotation has also changed from a single focus on physical health to a broader concept of overall health (2). Although wellness tourism has accumulated a wealth of experience in long-term practice, in the complex and changing realities of the situation, it is still facing multiple challenges such as the impact of public health emergencies (e.g., COVID-19), increased vulnerability of the ecological environment, and structural changes in consumer demand, and there exists a risk of “chain breakage” (3), There is an urgent need to reconstruct the industrial resilience system through the application of digital recreation technology, green service model innovation, human capital quality enhancement and other NQPF's elements (4). NQPF, the most recent theoretical development resulting from the adaptation of Marxism to the Chinese context, has profound implications and a rich vocabulary (5). The word “new” refers to “innovation” at the technological level, with state-of-the-art technology serving as the driving force behind productivity in the “new” areas, which in turn releases the momentum of development; “quality” emphasizes quality, which suggests a higher level of development and a better standard of living. Different from the linear superposition of traditional productivity factors, the NQPF's theory emphasizes the organic combination of “new-type laborers”, “new-type labor tools”, and “new-type labor objects”, which can bring more development space for the wellness tourism industry and bring more space for development (6).

We take 30 Chinese provinces and municipalities as the research object in order to thoroughly examine the role that NQPF plays in influencing the resilience of the wellness tourism industry. We then use multi-source data to measure the resilience of wellness tourism and the degree of development of NQPF in China, with the aim of answering the following four questions:

1. What kind of temporal and spatial distribution best describes the resilience of wellness tourism in China?
2. Does the industrial resilience of wellness tourism benefit from NQPF?
3. Does the human capital aggregation effect of NQPF contribute in a non-linear way to the industrial resilience of wellness tourism?
4. How can the creation and modification of current policies support the general upgrading of the wellness tourism sector to NQPF?

## 2 Literature review and research hypotheses

### 2.1 Synthesis study

NQPF has emerged as a major force behind fundamental shifts in the industrial development model, economic structure, and mode of production in the age of scientific and technological innovation (7). The NQPF's theoretical framework exhibits a multifaceted and interdisciplinary research feature. According to Shan and Han (8). NQPF is a new kind of productivity that deviates from conventional routes, depends on disruptive technologies, and is intricately linked with the digital economy. This conceptualization has received extended validation from Ren et al. (9), who expanded the analytical scope to industrial practices by systematically elucidating the intrinsic logic of NQPF in driving banking competition through revolutionary technological breakthroughs, allocation of innovative factors, and industrial upgrading. This advancement significantly updates scholarly understanding of NQPF's sector-specific impacts. Building on this conceptualization, Shan et al. (8) develop a measurement framework that incorporates both substantive and pervasive elements, and their empirical validation explores the potential regional synergies between digitization and greening in fostering the development of NQPFs and promoting high-quality growth. However, critical gaps persist in contemporary research. Despite the robust growth momentum of modern service sectors like wellness tourism. The explanatory capacity and guiding value of NQPF theory for the superior development of contemporary service industries are severely limited by this structural mismatch between theoretical constructs and industrial practices.

The wellness tourism industry's distinct reliance on services, ecological reliance, and health orientation aligns in many ways with NQPF's green, digital, and human-centered characteristics. Three main theoretical panels on wellness tourism have been established by academics. The first panel focuses on the industry's ecological benefits and how to exploit them. The effectiveness of many natural ecological resources in promoting wellness is a key tenet of wellness tourism (10, 11). Because of the close ties between these ecological environments and regional cultural traditions, the industry's ability to develop can be greatly increased by utilizing and transferring these ecological cultures (12). But there are drawbacks to this reliance as well. Because the ecological environment is so delicate, any disruption to environmental conditions could result in systemic risks, and the long-term growth of the wellness tourism sector cannot be achieved by solely depending on the environment. The second one focuses on the reasons behind consumer demand and how the wellness tourism sector is changing. This theoretical panel confirms the critical role those various stakeholders (such as travel and hospitality providers) play in ensuring that travelers have a positive travel experience (13, 14). It is important to keep in mind that various wellness tourism groups have varying reasons for wanting to travel, and the creation of unique service approaches is an unavoidable trend in the growth of the wellness tourism sector (15). Aggregation of human capital will be the primary assurance for the unique growth of the wellness tourism sector in such a scenario (16). Excellent human resources will produce a more complete service system that can accommodate various customer needs. Thirdly, a study on the distribution of wellness tourism resources found that the effect of wellness tourism is mainly concentrated in economically developed areas with rich resources and strong support ability (17). This implies that NQPF's enabling role will vary depending on the region and the resources available for wellness tourism. Therefore, it is essential to further elucidate NQPF's enabling role in regions with varying levels of development in order to support the creation of differentiated development strategies with empirical data.

Even though the aforementioned research findings have created a broad framework for the field of wellness tourism, the current body of research remains limited to the analysis of static elements, exhibiting a certain theoretical lag and failing to address the central claim of NQPF reconfiguring the mechanism of industrial resilience. The following three elements primarily demonstrate the study's theoretical worth. First, we construct the theoretical framework of “three-dimensional empowerment and four-dimensional evaluation”, providing objective standards for measuring NQPF and wellness tourism resilience. Second, we visualize the distribution of spatial and temporal patterns of wellness tourism resilience and NQPF, revealing the differences in characteristics between the two at different levels of regional development and different levels of industrial maturity. Third, human capital agglomeration is introduced as a key threshold variable to reveal the moderating effect of human capital agglomeration on the authorization threshold of NQPF, which offers theoretical support for the development of precise industrial policies.

### 2.2 Research hypotheses

#### 2.2.1 NQPF's direct impact on wellness tourism resilience

NQPF is a representative of contemporary advanced productivity in the combination of labors, labor objects and labor tools (18), The industrial resilience's security threshold and competitive resilience are greatly enhanced by this productivity system through the formation of strong industrial partnerships. In this section, we will reveal the mechanism of NQPF on wellness tourism resilience from the perspective of factor deconstruction.

As the most active element of NQPF, new-type labors offer intellectual support for industrial resilience due to their higher educational attainment and stronger professional knowledge base. Multidisciplinary skills can be applied synergistically in wellness tourism service scenarios by new practitioners who possess interdisciplinary knowledge systems and digital transformation capabilities. This structural upgrading of human capital not only optimizes the integrated service process of “medical care and tourism”, but also improves the response efficiency to market fluctuations through a dynamic knowledge-sharing mechanism.

The value reconstruction of new-type labor objects strengthens the industry chain's ability to resist risks. Non-physical labor objects with more technological components, including data and information, are also directly generating significant economic value under the ongoing iterations of science and technology. These factors, which include high value density and strong demand orientation, compel industrial subjects to set up a precise demand response mechanism and a flexible system for product research and development in order to effectively adapt to changes in the market.

The way the wellness tourism sector operates has changed as a result of the clever adaptation of new-type labor tools. The way the wellness tourism sector operates has changed as a result of the clever adaptation of new-type labor tools. Conventional physical manufacturing items can be elevated to the digital level through the thorough integration of digital and conventional labor methods. In addition to achieving the transition from traditional service delivery to the full health management process, this transformation effectively addresses the industrial chain's “broken chain” risk and synergistic dilemma through innovations such as supply chain visualization and service process standardization. Together, the aforementioned components' synergistic innovation forms the response mechanism of NQPF empowering wellness tourism resilience, offering a fresh viewpoint on the industry's sustainable growth. Based on this, this study proposes the first core hypothesis:

H1: NQPF can significantly increase the level of resilience of the wellness tourism chain.

#### 2.2.2 The mediating effect of industrial structure upgrading

The empowerment effect of NQPF on health tourism resilience shows a multidimensional mechanism. With its core advantages in resource allocation optimization and industrial collaborative innovation, new productivity has effectively promoted the evolution of health tourism industry structure to high-level and rationalization, thus, it significantly improves the system resilience of the industry to cope with external challenges and shocks. The upgrading of industrial structure has promoted the development of personalized health tourism services, and has greatly extended and supplemented the service boundaries of the traditional medical system. On this basis, we put forward the second core hypothesis.

H2: NQPF empowers the resilience of wellness tourism by optimizing the industrial structure.

#### 2.2.3 Threshold effects of human capital agglomeration

As traditional industry clusters, cross-industry clusters, and emerging industries mature, the knowledge spillover effect created by talent concentration has emerged as the primary driver for industry cluster upgrading. Previous research has demonstrated that human capital is the primary determinant of collaborative innovation in the industry chain. The breadth and depth of knowledge sharing and technology diffusion are constrained by the industry's lack of human resources, and the knowledge spillover effect and innovation capacity enhancement are comparatively limited when human capital aggregation is low (19), Wellness tourism industry is difficult to quickly adjust and cope with external shocks, and is prone to development bottlenecks. When human capital reaches a certain level, however, the concentration of top-notch talent propels the improvement of the knowledge spillover effect and continuously encourages the quick spread of knowledge and the iterative updating of technology, which supports the industry's synergistic growth and resource allocation optimization. This nonlinear threshold effect reveals that there may be significant stage heterogeneity in the enabling effect of NQPF on chain resilience. Based on this, we propose the second hypothesis:

H3: After crossing the threshold of human capital agglomeration, NQPF's impact on wellness travel industrial resilience will be defined by growing marginal benefits.

## 3 Methodology

### 3.1 Model

#### 3.1.1 Benchmark regression model

Referring to Hausman J (20), we performed a Hausman test, which revealed significant differences between the estimates from the random effects and fixed effects models(Prob > chi2 = 0.0000), Therefore, in order to improve the accuracy of the model, we use a double fixed-effects model controlling for time and region to our regression analysis.

(1) $$WT_{it}=\beta_{0}+\beta_{1}Npqf_{it}+\beta_{2}Controls_{it}+\eta_{i}+\delta_{t}+\varepsilon_{it}$$

Where the subscripts *i* and t denote region and year, respectively, and the core explanatory variable $Npq_{it}$ denotes the level of NQPF from region i in year *t*, $WT_{it}$ denotes the level of wellness tourism resilience of the region *i* in the period $t,\;Controls_{it}$ denotes the group of control variables, $\beta_{0}\;$ is a constant term, we focused on the coefficient $\beta_{1}$, which denotes the marginal effect of NQPF on wellness tourism resilience, If $\beta_{1}$ is significantly positive, it indicates that NQPF has a positive promotion effect on wellness tourism resilience. $\eta_{i}$ and $\delta_{t}$ were used to control for individual fixed effects and time fixed effects, respectively, $\varepsilon_{it}\;$ is the random error term.

#### 3.1.2 Mediation effect model

To explore the potential mechanisms through which NQPF may influence the resilience of wellness tourism, this study builds upon the baseline model (1) and employs the mediation effect approach to construct a mediation model. Specifically, given the statistical significance of coefficient $\beta_{1}$ in Equation 1, we subsequently establish two additional equations: Equation 2 examines the effect of new quality productive forces $Npqf_{it}$ on the mediating variable Indu, while Equation 3 examines the joint influence of both $Npqf_{it}$ and the mediating variable Indu on $WT_{it}$. This methodological approach allows for systematic testing of whether and to what degree industrial structure upgrading functions as an intermediary channel through which NQPF enhance the resilience of the wellness tourism sector.


(2) $$Indu_{it}=\alpha_{0}+\alpha_{1}Npqf_{it}+\alpha_{2}Controls_{it}+\eta_{i}+\delta_{t}+\varepsilon_{it}$$

(3) $$WT_{it}=\gamma_{0}+\gamma_{1}Npqf_{it}+\gamma_{2}Indu_{it}+\gamma_{3}Controls_{it}+\eta_{i}+\delta_{t}+\varepsilon_{it}$$

#### 3.1.3 Threshold effect model

Our analysis in section 2 suggests that human capital agglomeration may have a nonlinear threshold effect on NQPF-enabled wellness tourism resilience. In order to investigate the dynamic change characteristics of human capital agglomeration in fostering industrial resilience at various stages of development and to further validate this theoretical hypothesis, We refer to the threshold regression method proposed by Hansen (21) to construct the theoretical model. As shown in Equation 4, this model uses human capital agglomeration HC as the core threshold variable, and constructs a segmented regression equation by introducing an indicator function I(⋅), where $\lambda_{i}$ denotes the threshold of significance estimated by the Bootstrap method, $\tau_{i}$ indicates the degree of influence of NQPF on wellness tourism resilience at different thresholds. Through stratification and testing for the presence of multiple threshold effects, the model offers a quantitative analytical framework for comprehending the stage-specific effects of human capital aggregation. Not only can we determine the human capital agglomeration optimization interval, but we can also give local governments a theoretical foundation on which to build unique HR allocation policies.


(4) $$\begin{array}{c} WT_{it}=\tau_{0}+\tau_{1}Nqp_{it}*I(\mathrm{HC}\leq \lambda_{1})+\tau_{2}Nqp_{it}*I(\lambda_{1}<HC\leq \lambda_{2})\;\\ \;+\cdots +\tau_{n}Nqp_{it}*I(\lambda_{n-1}\left\langle \mathrm{HC}\leq \lambda_{n})+\tau_{n-1}Nqp_{it}*I(HC\right\rangle \lambda_{n})\;\\ \;+\eta_{i}+\delta_{t}+\varepsilon_{it}\; \end{array}$$

### 3.2 Variable definitions and descriptions

#### 3.2.1 Independent variable

This study builds the NQPF evaluation index system from the three dimensions of New-type laborers, New-type labor objects and New-type labor tools (Table 1) based on the fundamental ideas of Marxist political economy. Simultaneously, we present the coupled coordination model to compute the synergistic effect of production factors, creating a theoretically sound and practically useful analytical framework.

**Table 1:** Evaluation Index system for the NQPF.

Primary indicator	Secondary indicator	Tertiary indicator	Direction
New-Type Laborers	Laborer Skills	Per Capita Years of Education	▴
		Full-time R&D Personnel Equivalent in Industrial Enterprises Above Designated Size	▴
		R&D/GDP	▴
	Laborer Productivity	Average Wage per Employee	▴
		Technology Market Transaction Value	▴
	Laborer Awareness	Employment Share in Tertiary Sector	▴
New-Type Labor Objects	Emerging Industries	Output Value of High-tech Industries	▴
	Future Industries	Robot Installation Density	▴
		Domestic Utility Model Patents Granted	▴
	Ecological Environment	Forest Coverage Rate	▴
		Environmental Protection Expenditure Ratio	▴
New-Type Labor Tools	Digital Infrastructure	Internet Broadband Access Ports	▴
		Optical Cable Line Length	▴
	Digitalization Level	Digital Economy Development Index	▴
		Enterprise Digital Maturity Index	▴
	Energy Efficiency	Energy Consumption per Unit GDP	▾

▴ Positive indicator (higher value preferred) ▾ Negative indicator (lower value preferred).

The effectiveness of productivity development is directly impacted by the competence and quality of workers, who are the dynamic subjects of the productivity system. We measure New-type laborers in terms of laborer skills, laborer productivity, and laborer awareness. The skill dimension was selected to characterize educational level and R&D efforts; By combining the average wage per employee, which represents labor input, with the turnover rate in the technology market, the laborer productivity dimension calculates the extent to which scientific and technological advancements have been translated into real productivity; The percentage of persons employed in the tertiary sector was selected to measure the laborer awareness.

We build a assessment system for emerging industries, future industries, and the ecological environment in response to the broadening of the scope of traditional labor objects. High growth, high value-added, and high technological content are characteristics of emerging industries. The output value of high-tech industries is chosen for measurement, and it is computed using the funds allocated for the development of new products (22); The future of the industry is forward-looking and potentially valuable to the market, as measured by the installed density of industrial robots and the number of patents on artificial intelligence. The choice of two indicators—forest cover rate and the proportion of money spent on environmental protection—to gauge the state of the ecological environment (23). In order to break through the perceived boundaries of traditional means of production, three dimensions of digital infrastructure, digitization level and energy efficiency were selected to measure the new-type labor tools (22). Fiber-optic cable line length and internet penetration were chosen as indicators of digital infrastructure; Digital economy development index and enterprise digital maturity index were chosen to indicate the level of digital transformation; the renewable energy consumption per unit of GDP was used to characterize the proportion of renewable energy use and the degree of development of green economy (24).

Since the synergistic effect of multiple elements is the fundamental source of NQPF, We builds a coupled coordination model to quantify the degree of NQPF development in terms of the degree of coupled coordination among subsystems. The model can systematically reflect the dynamic coupling relationship between technological revolution breakthroughs and elemental innovation allocation, and it successfully overcomes the limitation of fragmentation of elements in traditional evaluation methods (25).

(5) $$C=3\times [nlp\times nol\times nmp/nlp+nol+nmp^{3}{]}^{1/3}$$

(6) T=ϕnlp+φnol+δnmp

(7) $$Nqp=\sqrt{C\times T}$$

Equation 5 is the subsystem's coupled coordination degree, nlp, nol and nmp are the T-scores of New-type laborers, New-type labor objects, and New-type labor tools, respectively, Equation 6 is the integrated and coordinated development index of NQPF, assuming that laborers, labor objects, and labor materials are equally important, and taking ϕ=φ=δ=1/3. Nqp is the development level of NQPF in Equation 7.

#### 3.2.2 Dependent variable

The wellness tourism industry represents a highly integrated and multifaceted industrial sector, inherently encompassing various specialized segments such as medical tourism, hot spring therapy, forest wellness retreats, traditional Chinese medicine-based wellness programs, and elderly care tourism. The developmental efficacy of this industry is synergistically influenced by multidimensional factors including infrastructure, social resources, and market conditions. Currently, academia has yet to establish a unified standard system for assessing resilience in wellness tourism.

To address this gap, this study synthesizes cutting-edge international academic research (26–28) with the objective of extracting common characteristics across diverse wellness tourism subcategories. We have developed a comprehensive evaluation framework comprising four key dimensions—economic resilience, social resilience, environmental resilience and adaptive resilience—encompassing a total of 24 indicators (Table 2). We use the TOPSIS method for objective assignment and uses the information entropy to determine the weight coefficients of each indicator. This ensures that the weight distribution is objective. In addition to taking into account the actual financial outlays of the wellness tourism sector as well as its market share, economic resilience evaluates the stability and capacity for recovery of the sector during periods of economic upheaval and crisis; The degree of aging, the extent of medical insurance coverage, the level of transportation development, and the maturity of regional services are the four factors we use to evaluate the wellness tourism industry's responsiveness to social needs and adaptability to social changes; We chose resource abundance and pollution treatment capability to define environmental resilience, which measures the wellness tourism sector's capacity to adapt and bounce back from outside ecological stresses; The provision of healthcare public services and labor resources are included in the Adaptive Resilience dimension, which shows how adaptable the sector is to a range of changes and uncertainties.

**Table 2:** Evaluation Index system for the wellness tourism industry resilience.

Primary indicator	Secondary indicator	Tertiary indicator	Direction
Economic Resilience	Operating Income	Tourism Revenue	▴
	Resource Endowment	Number of Tourist Attractions	▴
	Economic Structure	Percentage of Total Health Expenditure to GDP	▴
	Fiscal Expenditure	Local Fiscal Expenditure on Healthcare	▴
Social Resilience	Elderly Care Services	Old-age Dependency Ratio	▴
	Consumption Level	Consumer Price Index	▴
	Medical Security	Year-end Urban Medical Insurance Participants	▴
	Population Density	Urban Population Density	▴
	Cultural Services	Number of Rural Cultural Towns	▴
	Transportation	Passenger Traffic Volume	▴
	Reception Capacity	Number of Travel Agencies	▴
Environmental Resilience	Waste Management	Domestic Waste Harmless Treatment Capacity	▴
	Sewage Treatment	Urban Sewage Treatment Rate	▴
	Livability Endowment	Annual Average Temperature	▴
		Urban Green Space Coverage	▴
		Annual Average Relative Humidity	▴
		Number of Parks	▴
Adaptive Resilience	Medical Facilities	Number of Healthcare Institutions	▴
		Number of Rehabilitation Medicine Beds	▴
		Number of Hospital Beds per 10,000 Population	▴
	Public Services	Public Administration and Service Land Area	▴
	Human Resources	Number of Licensed Physicians	▴
		Number of Health Technical Management Personnel	▴
		Number of Travel Agency Employees	▴

▴ denotes positive indicator (higher value indicates better performance).

#### 3.2.3 Mediating variable

The rationalization of industrial structure reflects the efficiency of coordination among industries during the economic development of a country or region. It involves adjusting and optimizing the proportional relationships and resource allocation among different sectors within industries to achieve more harmonious and efficient operations. Therefore, this study employs the industrial structure rationalization index to measure industrial structure upgrading, calculated as shown in Equation 8. where $y_{i}$ denotes the proportion of value-added from the *i* industry to GDP.


(8) $$Indu=\sum_{i=1}^{3}(i\times y_{i})=1\times y_{1}+2\times y_{2}+3\times y_{3}$$

#### 3.2.4 Threshold variables

Human capital serves as a pivotal driver for industrial structure transformation, facilitating regional innovation ecosystems, optimizing resource allocation efficiency, and enhancing modern management systems. These synergistic effects collectively promote the coordinated development of industrial clusters while strengthening their risk resilience. Following established methodologies in spatial economics, this study employs the location entropy index of urban populations holding associate degrees or higher as the primary metric for quantifying human capital agglomeration.

(9) $$Hc_{it}=\frac{EC_{it}/E_{i}}{EC_{t}/E_{t}}$$

In Equation 9, $EC_{it}$ denotes the total number of persons with tertiary education or above in the period; $E_{i}$ denotes the total number of employed persons in the region; $\;EC_{t}$ and $E_{t}$ represent the total number of tertiaries educated individuals and the total number of employed individuals in the nation during period t, respectively.

#### 3.2.5 Control variables

To ensure empirical robustness, the following control variables were incorporated to account for potential confounding effects:

1. Government Involvement (Gov): Measured by the ratio of local fiscal expenditure to regional GDP;
2. Marketization Level (Mar): Operationalized using the provincial marketization index sourced from the Marketization Index Report by Chinese Provinces (2021);
3. Urbanization Rate (Urb): Calculated as the proportion of urban district population relative to the prefecture-level city's total population;
4. Trade Openness (Open): Defined as the share of total import-export volume in regional GDP;
5. Economic Development (LnGDP): Represented by the natural logarithm of regional gross domestic product.

### 3.3 Data sources and descriptions

This study utilizes panel data from 30 Chinese provinces/municipalities during 2014–2022 (excluding Tibet Autonomous Region, Hong Kong, Macao, and Taiwan due to severe data gaps). Original data were compiled from authoritative sources including *China Statistical Yearbook*, *China Health Statistical Yearbook*, *China Statistical Yearbook on Science and Technology*, *China Industrial Statistical Yearbook*, provincial statistical yearbooks, official statistical bulletins, and the CSMAR database. Missing values were addressed using linear interpolation methodology to ensure data continuity.

Table 3 presents descriptive statistics for key variables across 270 observations. The core explanatory variable, NQPF development level, demonstrates substantial variation with maximum (0.7976), minimum (0.1969), mean (0.3901), and standard deviation (0.1077), indicating significant spatial-temporal disparities in China's productivity advancement. The dependent variable, resilience of wellness tourism, exhibits a wide range from 0.0481 to 0.7007 (mean = 0.2646, SD = 0.1307). The observed distribution patterns. These statistical characteristics reinforce the methodological necessity of controlling for regional heterogeneity in our empirical framework.

**Table 3:** Descriptive statistical analysis.

Type	Variable	Obst	Mean	Std. Dev.	Min	Max
Dependent Variable	WT	270	0.2646	0.1307	0.0481	0.7007
Core Explanatory Variable	NQPF	270	0.3901	0.1077	0.1969	0.7976
Control Variables	Gov	270	0.2601	0.1091	0.1050	0.7534
	Mar	270	8.1852	2.1092	3.0700	16.7100
	Urb	270	0.6202	0.1104	0.4025	0.8933
	Ope	270	0.1081	0.1499	0.0001	0.8787
	LnGDP	270	9.9850	0.8765	7.5217	11.7715
Threshold Variable	HC	270	1.1078	0.5013	0.3520	4.3662

## 4 Results

### 4.1 Spatial distribution characteristics

**Figure 1:**
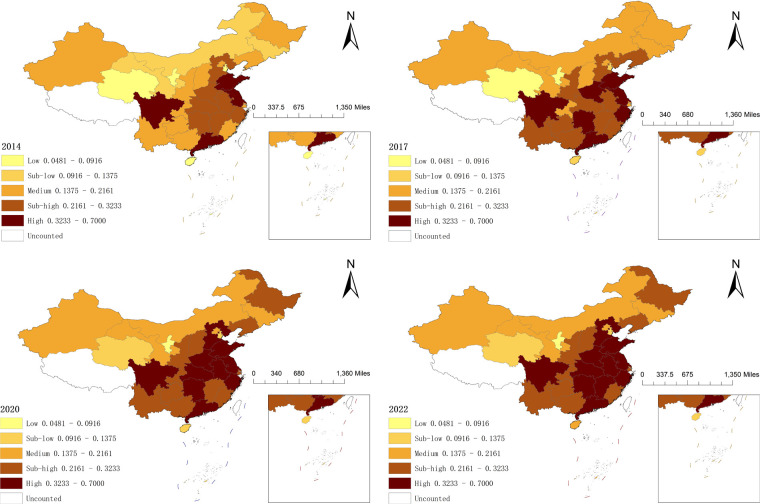
Provincial spatial evolution of wellness tourism resilience. Map lines delineate study areas and do not necessarily depict accepted national boundaries.

**Figure 2:**
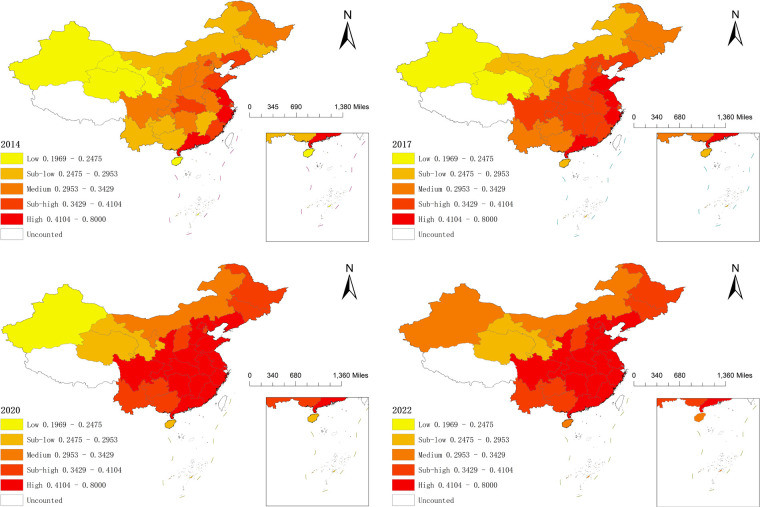
Provincial spatial evolution of NQPF. Map lines delineate study areas and do not necessarily depict accepted national boundaries.

We classified the multi-period data from 2014 to 2022 into a five-level gradient using the Arc-GIS 10.8 natural breakpoint method based on the level of resilience of wellness tourism (Figure 1) and the level of NQPF measure (Figure 2).

All of China's regions exhibit resilience in wellness tourism, with “high in the south and low in the north” distribution characteristics. 2014 established a spatial framework of “four cores and seven axes”, with the high resilience core area consisting of Shandong, Jiangsu, Guangdong, and Sichuan, and the sub-high resilience zone consisting of the seven central Chinese provinces, where the wellness tourism sector began earlier and where there is adequate human capital and livable climate. A low resilience agglomeration can be seen in the northwest, which includes the four provinces of Qinghai, Gansu, Inner Mongolia, and Jilin. These regions have arid climates and lack the natural environmental base that the wellness tourism sector depends on. Four provinces—Shaanxi, Guizhou, Yunnan, and Guangxi—saw an increase in resilience in 2017, and the center of gravity of the wellness tourism sector gradually moved from the coastal region to the inland area. Zhejiang and Hunan were ranked among the high resilience zones in 2017. As the northern region's representative province, Heilongjiang initially achieved sub-high resilience in 2020. The country's 2022 wellness tourism resilience level spatial pattern has been initially established, with high resilience areas primarily located in the southwestern and eastern regions, including six provinces: Sichuan, Shandong, Henan, Guangdong, Jiangsu, and Zhejiang. These areas have improved the wellness tourism industry's capacity to withstand the effects of external risks due to their robust scientific and technological innovation foundation, robust economies, easy access to transportation, and larger talent pools. The eastern and some central regions, such as Hubei, Anhui, Hunan, and other provinces and cities, are home to sub-high toughness zones. Because of their high basic productivity, these areas have developed a more stable wellness tourism industry chain. The medium toughness zone includes the provinces of Shanxi, Gansu, Chongqing, Guizhou, and Inner Mongolia, whereas the second-low toughness zone and the low toughness zone are mainly located in the western and northern regions, where the growth of the tourism industry is limited by altitude, terrain, and economic development.

The nation's NQPF level demonstrates a three-stage development path of “Coastal Leadership-Central Rise-Regional Synergy”. Only the Yangtze River Delta and Pearl River Delta regions reached a high level of NQPF in 2014, while six provinces—Shanghai, Beijing, Shandong, Hubei, Fujian, and Liaoning—attained a second-high level. The country's overall level of NQPF development was low at the time, but the southeast coastal region had already established a “prototype” of high-level agglomeration and NQPF development. Shandong was one of the high-level regions in 2017, with nine provinces and cities, including Sichuan and Shandong, improving to varied degrees. Twelve provinces, including Beijing, Sichuan, Anhui, and Jiangxi, have been promoted to high level development regions, while the majority of the cities and provinces along the southeast coast have already jumped to the medium level and above in 2020. Some central regions have also increased their level of NQPF development. With the provinces' ranking order staying constant in 2022, the map of NQPF development now enters a phase of dynamic equilibrium.

### 4.2 Baseline regression results

Table 4 presents systematic robustness checks through progressive model specifications. Column (1) establishes baseline estimates with comprehensive controls under both time and entity fixed effects. Furthermore, Columns (2)–(4) demonstrate remarkable stability of the core explanatory variable's coefficients, maintaining a range of 0.278 to 0.375 across alternative model specifications (with/without control variables and time fixed effects), all statistically significant at the 1% level (*p* < 0.01). These consistent findings provide robust empirical validation for theoretical hypothesis H1, confirming that the advancement of NQPF significantly enhances systemic resilience in the wellness tourism industry.

**Table 4:** Baseline regression results.

Variables	(1) WT	(2) WT	(3) WT	(4) WT
NQPF		0.394*** (11.904)	0.259*** .747)	0.317*** (8.263)
Gov	0.281*** (5.326)		0.141*** (2.966)	0.159*** (3.259)
Mar	0.003*** (2.728)		0.001 (0.837)	0.000 (0.196)
Urb	0.551*** (4.880)		0.376*** (4.225)	0.454*** (4.555)
Ope	−0.172*** (−4.795)		−0.070** (−2.234)	−0.085** (−2.541)
LnGDP	0.036 (1.581)		0.043** (2.474)	0.006 (0.291)
_cons	−0.517** (−2.500)	0.084*** (7.608)	−0.546*** (−4.113)	−0.236 (−1.277)
N	270	270	270	270
R^2^	0.864	0.880	0.869	0.896
F	111.344	188.048	258.287	138.913
Individual fixed effects	Yes	Yes	No	Yes
Time fixed effects	Yes	Yes	Yes	Yes

*, **, and *** indicate significant at the 10%, 5%, and 1% levels, respectively; values in parentheses are t-test statistics. Same as below.

### 4.3 Robustness tests

We conducted comprehensive robustness checks through three methodological adjustments. First, recomputing NQPF using the entropy-weighted TOPSIS method instead of the original coupling coordination degree yielded a consistent positive coefficient, as shown in Column (1) of Table 5. Second, to address jurisdictional heterogeneity arising from the unique policy advantages and infrastructural differences of Beijing, Tianjin, Shanghai, and Chongqing, we exclude these cities, observing a slightly stronger effect that is still statistically robust (Column 2). Third, in order to mitigate the bias of the results due to the effect of outlier points, all continuous variables in this study were subjected to 1% bilateral shrinkage and regressed again, and the results are shown in Column (3). The validity of the previous findings is demonstrated by the noteworthy enabling effect of NQPF on the resilience of wellness tourism, which is significant across all three robustness tests.

**Table 5:** Robustness test results.

Variables	(1) WT	(2) WT	(3) WT
NQPF	0.301*** (9.461)	0.321*** (7.515)	0.309*** (8.034)
Gov	0.162*** (3.472)	0.157*** (2.744)	0.172*** (3.358)
Mar	−0.001 (−1.206)	0.000 (0.071)	0.000 (0.322)
Urb	0.541*** (5.658)	0.415** (2.550)	0.363*** (4.063)
Ope	−0.048 (−1.440)	−0.070 (−1.641)	−0.047 (−1.452)
LnGDP	0.013 (0.667)	0.010 (0.415)	0.015 (0.812)
_cons	−0.278 (−1.565)	−0.233 (−1.093)	−0.283 (−1.609)
N	270	234	270
R2	0.903	0.898	0.894
F	150.098	121.424	136.866

### 4.4 Mediation effect tests

The preceding theoretical analysis has examined the transmission mechanism through which NQPF empower the resilience of the wellness tourism. We now proceed to conduct empirical verification using a mediation effect model, with the regression results presented in Table 6.

**Table 6:** Mediation effect test.

Variables	(1) Indu	(2) WT
NQPF	0.231*** (4.175)	0.280*** (7.114)
Gov	0.300*** (4.065)	0.135** (2.568)
Market	0.000 (0.199)	0.000 (0.290)
Urban	0.236* (1.837)	0.333*** (3.756)
Open	−0.037 (−0.787)	−0.042 (−1.327)
LnGDP	−0.036 (−1.348)	0.020 (1.067)
Indu		0.126*** (2.762)
_cons	2.434*** (9.622)	−0.590*** (−2.864)
N	270	270
R2	0.733	0.898
F	44.368	131.996

The results have demonstrated that NQPF exert a significantly positive impact on the resilience of the wellness tourism industry, thereby satisfying the prerequisite for mediation effect analysis. The results in column (1) indicate that NQPF promote industrial structure upgrading, with a regression coefficient of 0.231 that is significantly positive at the 1% level. Column (2) shows that both NQPF and industrial structure upgrading have significantly positive coefficients at the 1% level. These findings suggest that driving industrial structure upgrading serves as a crucial pathway through which NQPF enhance the resilience of the wellness tourism industry, thereby providing validation for Hypothesis H2.

### 4.5 Endogeneity tests

We selected the instrumental variable two-stage least squares (2SLS) for robustness testing because of the possibility that endogeneity bias could be triggered by bidirectional causal associations. We employ the first-order lag term of NQPF (L.NQPF) as the instrumental variable (IV), with its validity grounded in two theoretical rationales. Firstly, the development trajectory of NQPF exhibits significant path dependence, as evidenced by the strong correlation between current and preceding productivity levels. Secondly, the one-period lagged specification effectively mitigates potential reverse causality concerns while enhancing exogenous characteristics. Table 7 column (1) demonstrates that this instrument successfully passes the first-stage regression test at the 1% significance level. Subsequent diagnostic tests further validate its appropriateness: The Kleibergen-Paap LM statistic of 46.330 (*p* = 0.0000) decisively rejects the null hypothesis of under identification, and the Kleibergen-Paap Wald F statistic of 205.650 substantially exceeds the 10% Stock-Yogo critical value threshold of 16.38, thereby confirming the absence of weak instrument issues. As shown in column (2), the second-stage estimation results maintain the statistically significant impact of NQPF on the resilience of wellness industry after addressing endogeneity concerns, reinforcing the robustness of our findings.

**Table 7:** Endogeneity tests.

Variables	(1) NQPF	(2) WT
L.NQPF	0.9235*** (14.34)	
NQPF		0.8274*** (14.15)
Constant	−0.1087* (−1.76)	−0.7707*** (−9.31)
Kleibergen-Paap LM		46.330 [0.0000]
Kleibergen-Paap Wald F		205.650 [16.38]
Control variables	Yes	Yes
Individual fixed effects	Yes	Yes
Time fixed effects	Yes	Yes
Observations	240	240
R-squared		0.914

### 4.6 Heterogeneity tests

We examine the heterogeneity of the impact of NQPF on wellness tourism resilience from three perspectives: the degree of economic development, the spatial geographic location, and before and after the policy's implementation. This will help to make the findings more broadly applicable.

#### 4.6.1 Economic heterogeneity analysis

The 30 provinces in this study are divided into underdeveloped and developed regions based on the mean annual GDP per capita in order to minimize the error caused by the uneven economic level on the regression results. The results are displayed in Table 8, columns (1)–(2). At the 1% level, NQPF has a significant positive impact on the resilience of wellness tourism in both developed and underdeveloped regions. The impact is greater in developed regions than in underdeveloped ones, which may be because highly developed regions have more abundant economic resources, residents with higher purchasing power, and a more active wellness tourism market, all of which enhance the empowering effect of NQPF.

**Table 8:** Heterogeneity test results.

Variables	(1) WT	(2) WT	(3) WT	(4) WT	(5) WT	(6) WT
NQPF	0.198*** (4.097)	0.445*** (5.072)	0.526*** (8.483)	0.190*** (3.718)	0.047 (0.279)	0.139*** (3.554)
Gov	0.193*** (3.067)	0.343*** (2.807)	0.088 (1.102)	0.225*** (3.028)	0.010 (0.110)	0.231*** (4.042)
Mar	0.000 (0.282)	−0.004* (−1.729)	−0.004** (−2.389)	0.002 (1.364)	−0.001 (−0.303)	0.001 (0.644)
Urb	0.350* (1.935)	0.417*** (3.136)	0.414*** (3.635)	0.416* (1.905)	0.111 (0.427)	0.415*** (3.438)
Ope	0.116* (1.733)	−0.009 (−0.187)	0.041 (0.988)	−0.018 (−0.182)	0.054 (0.833)	−0.177*** (−3.353)
LnGDP	0.038 (1.606)	−0.079* (−1.945)	−0.008 (−0.311)	0.066** (2.212)	0.089 (1.682)	0.019 (0.847)
_cons	−0.462** (−2.246)	0.575 (1.449)	−0.152 (−0.599)	−0.775** (−2.571)	−0.729 (−1.380)	−0.301 (−1.405)
N	171	99	117	153	60	210
R^2^	0.891	0.929	0.925	0.889	0.687	0.863
F	80.314	69.567	79.403	70.091	7.209	88.505

#### 4.6.2 Regional heterogeneity analysis

Provinces also differ in how they have developed their industrial structure, market conditions, and resource allocation, as well as how NQPF has affected wellness tourism. Regional heterogeneity is another way to define industrial resilience. According to columns (3)–(4) in Table 8, NQPF significantly affects wellness tourism resilience at the 1% level in both the Eastern and Midwestern regions, with the Eastern region experiencing a greater impact.

#### 4.6.3 Heterogeneity analysis before and after policy implementation

A total of 49 policy documents were published in 2016, the first year that the number of wellness tourism policies peaked, and the former National Tourism Administration released the National Wellness Tourism Demonstration Base standard, establishing wellness tourism as a new type of tourism. In order to explore the impact of NQPF on the level of wellness tourism resilience before and after the implementation of the policy, we take 2016 as the limit to divide the sample, and 2014–2015 is the “emergence stage”, as shown in column (5) of Table 8, and at this time, the promotion effect of NQPF on the wellness tourism resilience is not significant; The period of 2016–2022 is the “climbing stage”, as shown in column (6); the regression coefficient is 0.139 and significantly positive at the 1% level, which indicates that the policy support and guidance promote the empowering effect of NQPF on wellness tourism resilience.

### 4.7 Threshold effect test

To investigate the nonlinear impact of NQPF on the resilience of wellness tourism industry, we employ human capital agglomeration (HC) as the threshold variable with 300 bootstrap replications. Table 9 presents the threshold effect test results: Both single and double thresholds pass the test at 1% significance level, with threshold values of 0.6737 and 0.7810, respectively, and the result of triple threshold is not significant, indicating that the degree of human capital agglomeration has only a double-threshold effect in NQPF affecting wellness tourism resilience. Table 10 reveals the regime dependence coefficient of NQPF, and the non-linear spillover of monotonic growth can be observed from the threshold effect of talent capital agglomeration. These findings confirm the H3 hypothesis regarding the threshold modulation of resilience enhancement.

**Table 9:** Threshold effect test.

Thrsh	Thrsh Val	Fstat	Prob	Crit10	Crit5	Crit11	BS Reps
(1) Thrsh	0.6737	45.48***	0.0000	16.1292	18.4324	23.0294	300
(2) Thrsh	0.7810	23.42***	0.0133	13.7921	17.0359	25.3402	300
(3) Thrsh	0.8338	5.400	0.7067	17.1007	18.7928	25.7047	300

**Table 10:** Threshold effect test results.

Variables	WT
NQPF (*HC* < 0.6737)	0.1861** (0.0683)
NQPF (0.6737 ≤ *HC* < 0.7810)	0.2320*** (0.0680)
NQPF (*HC* ≥ 0.7810)	0.2739*** (0.0631)
Control variables	Yes
Individual fixed effects	Yes
Time fixed effects	Yes
__cons	−0.3440*** (0.113)
R2	0.899
Sample size	270

## 5 Conclusions

### 5.1 Theoretical implications

We use panel data from 30 Chinese provinces and cities between 2014 and 2022 to systematically build the evaluation index system of NQPF and wellness tourism resilience. The temporal and spatial distribution patterns of wellness tourism resilience and NQPF are revealed respectively, and the empowering effect of NQPF on wellness tourism resilience is empirically tested by using the double fixed effect model and the threshold effect model. The main theoretical implications are as follows:

Firstly, both NQPF and wellness tourism resilience exhibit notable temporal and spatial evolution traits. Wellness tourism resilience system forms a five-level gradient structure: The southeast hills form the contiguous center of the high resilience zone [0.3233, 0.7], and the sub-high resilience zone [0.2161, 0.3233) is distributed in a band in North and Northeast China, the medium resilience zone [0.1375, 0.2161), the sub-low resilience zone [0.0916,0.1375), and the low resilience zone [0.0481, 0.0916) are decreasing in a gradient. NQPF is similarly divided into five types of areas: high level [0.4104, 0.8000), sub-high level [0.3429, 0.4104), medium level [0.2953, 0.3429), sub-low level [0.2475, 0.2953), and low level [0.1969, 0.2475] areas, its level of development shows a tendency to radiate and spread from the southeastern region to the central region and even nationwide. Due to geographical and other environmental factors, the infrastructure in the western regions is relatively weak, which constrains the development of NQPF-related elements. Although there are national-level regional coordination policies such as the Western Development Strategy, there are still gaps in the specific policy support for the integration of the health tourism industry and NQPF, the precision of resource allocation, and the efficiency of local implementation. This has resulted in the failure to fully convert policy dividends into development momentum, limiting their role in enhancing the resilience.

The benchmark regression results of the bidirectional fixed effects model indicate that NQPF significantly promotes the resilience of health tourism, and the endogeneity and robustness tests further confirm the reliability of this result. The rationality of industrial structure is an important pathway for new-type productivity to enhance the resilience of health tourism. New-type productivity promotes the transformation of traditional medical levels to high-value-added new formats such as precision medicine, forming an integrated industrial chain of “medical care—health maintenance—leisure”. This process of upgrading the industrial structure has significantly enhanced the supply quality and risk resistance capability of the health tourism system. This finding provides an important basis for formulating differentiated regional development policies: for the developed eastern regions, efforts should focus on strengthening the collaborative innovation between NQPF and high-end health tourism services; for the underdeveloped central and western regions, it is necessary to accelerate the rectification of the structural upgrade shortcomings in the health service industry through industrial policy guidance, thereby fully unleashing the potential benefits.

Thirdly, the resilience of wellness tourism and NQPF in terms of human capital aggregation have a double-threshold nonlinear relationship. When the level of human capital agglomeration is lower than the first threshold (0.6737), the contribution of NQPF to wellness tourism resilience is weak at 0.1861; When the level of human capital agglomeration exceeds the first threshold (0.6737) but is below the second threshold (0.7810), the coefficient of elasticity of empowerment jumps to 0.2320; When the second threshold is breached (0.7810), the elasticity coefficient increases to 0.2739 and the marginal effect stabilizes. This shows that there is a “gas pedal” effect of human capital accumulation, and only when it breaks through the critical scale, the empowering effect of NQPF on the resilience of wellness tourism can be fully released, highlighting the key regulatory function of human capital accumulation.

### 5.2 Managerial implications

This study offers a three-dimensional policy framework to guide the investigation of global wellness tourism industrial resilience and extracts practical lessons from China's development model based on empirical findings and international comparative analysis:

1. Building a “Digital Health Ecosystem”

NQPF is a high-level productive force led by scientific and technological innovation. With the rapid development of “Internet Plus”, China should take public health value as the guide, establish a development path combining the Wellness Tourism industry and digital technology, and create a smart wellness system integrating “prevention-intervention-rehabilitation”. Increase the rate at which the works, labor tools, and labor objects in the wellness tourism industry chain are transformed to meet NQPF standards. For instance, the development of a national health tourism digital pedestal that incorporates AI health risk assessment, wearable device real-time monitoring, and dynamic analysis of environmental health indices will make it easier for customers to combine entertainment and recuperation to create novel experiences.

2. Implementing the “Healthy Economy Circle”

The level of NQPF and wellness tourism resilience varies greatly across regions, so interregional variability should be considered when developing policies and local conditions should be taken into consideration. From a spatial perspective, the eastern region can try harder to combine the development paths of tourism, recreation, culture, and agriculture in a variety of industries; boost the productivity level of the health industry's technological innovation; fully utilize the multiplier effect of high-level regional agglomeration; and spread and propel the growth of the wellness tourism sector in other areas. The population advantage can be fully utilized by the central hub, which can also create a transit base for wellness services and offer preferential policies for talented workers to enhance the labor resources of wellness tourism. In order to support the transformation of ecological value, the resource-rich western regions can further integrate climate therapy and national medicine, create an international station for the rehabilitation of plateau diseases, develop a certification system for natural therapies like salt cave breathing diagnosis and treatment, and coordinate the creation of a trans-regional trading platform for health resource sharing.

3. Fostering a “New Health Talent Pipeline”

From the standpoint of modernizing public health governance, wellness tourism, as a key component of the Healthy China strategy, directly affects the caliber of its human capital, the effectiveness of the delivery of health services, and the development of public health resilience. The government can classify and put into action measures at the three levels of human capital introduction, training, and management to lessen the challenges that human capital faces during the mobility process. creating a strategic talent pool for health services, emphasizing the hiring of specialists in sports rehabilitation, geriatric care, and preventive medicine, and assisting in the establishment of a regional mobility subsidy program and cross-border accreditation of health professional credentials. encouraging the reform of the way that industry and education are integrated in the development of talent, as well as coordinating the talent mobility mechanisms in medical, recreational, and health care institutions as well as tourism institutions. Optimize the supply of talents for public health services for healthy aging by enhancing the capital density and specialization level of health service talents, so as to achieve the dual governance goals of high-quality development of the health industry and improvement of the health level of the whole population.

### 5.3 Limitations and future directions

There are still certain limitations to our study: the sample size is relatively small and includes provincial data from 2014 to 2022. It is challenging to uncover the diverse traits of market participants, even though provincial data can accurately depict macro-level operating patterns. In the following study, the unit of analysis is reduced to the micro level of the businesses, and the mechanism of NQPF's differential action across firms of different sizes, ownerships, and industries is thoroughly examined. Even though the study employs lagged one-period NQPF as an instrumental variable to address the endogeneity issue, potential omitted variables like the technological innovation environment and digital infrastructure could still interfere with causal identification. Causal inferences can then be strengthened in two ways: On the one hand, policy shock variables with exogenous nature are searched for; on the other hand, causal inference methods such as double-difference method and breakpoint regression are used to conduct more precise mechanism tests.

## Data Availability

Publicly available datasets were analyzed in this study. This data can be found here: https://data.csmar.com/ http://www.stats.gov.cn/sj/ndsj/.
